# An Investigation of the Outcomes of PGY Students’ Cognition of and Persistent Behavior in Learning through the Intervention of the Flipped Classroom in Taiwan

**DOI:** 10.1371/journal.pone.0167598

**Published:** 2016-12-02

**Authors:** Sheng-Der Hsu, Cheng-Jueng Chen, Wei-Kuo Chang, Yih-Jin Hu

**Affiliations:** 1 Division of General Surgery, Department of Surgery, Tri-Service General Hospital, National Defense Medical Center, Taipei, Taiwan, ROC; 2 Division of Trauma Surgery, Department of Surgery, Tri-Service General Hospital, National Defense Medical Center, Taipei, Taiwan, ROC; 3 Division of Gastroenterology, Department of Internal Medicine, Tri-Service General Hospital, National Defense Medical Center, Taipei, Taiwan, ROC; 4 Department of Health Promotion and Health Education, National Taiwan Normal University, Taipei, Taiwan, ROC; University of Westminster, UNITED KINGDOM

## Abstract

The Postgraduate Year (PGY) Program allows doctors-in-training to learn about the diagnosis, treatment and nursing of various common, general diseases. These items form the core curriculum and are mostly learned through caring for patients and clinical teaching. Doctors-in-training are evaluated for their knowledge through written tests or assignments, based on which the effectiveness of their training is also assessed; however, this generally produces a negative learning attitude among them. So we introduced the flipped classroom into PGY training program to change PGY students’ learning behavior. Although the flipped classroom is highly valued and has been practiced by teachers in schools of various levels, very few attempts have been made until now to report the learning outcomes achieved through the flipped classroom by means of rigorous research methods. Therefore we tried to employed Ajzen and Fishbein’s (1980) theory of reasoned action and Bandura’s self-efficacy to predict and explain the participants’ behavioral intention when participating in the core curriculum learning of the flipped classroom and to assess the change in students’ learning behavior and learning effectiveness. From August 2013 to July 2014, 39 PGY students from the General Surgery of the Tri-Service General Hospital were selected as the participants of this study. The control group included 43 students of the previous year, that is, the year before the intervention of the flipped classroom. A comparative analysis was performed. The questionnaire’s related matrices indicated highest correlation between self-efficacy and behavioral intention (r = 0.491, P < 0.01), followed by attitude (r = 0.365, P < 0.01) and subjective norms (r = 0.360, P < 0.01.) All three showed positive correlations with behavioral intention; among attitude, subjective norms, and self-efficacy, the pairwise correlations also reached significance level. The flipped classroom can indeed change PGY students’ the learning behavior from “passive learning” to “active learning.”

## Introduction

The Severe Acute Respiratory Syndrome (SARS) epidemic in 2003 revealed the years-old defects in Taiwan’s health care and medical education systems[1]. After the epidemic subsided, the Ministry of Health and Welfare proposed a plan to reform the training of clinical doctors and officially announced the implementation of the Postgraduate Year Medical Training Project for Training Doctors in Response to the SARS Epidemic in July 2003[2]. The project aimed to gradually rectify excessive and premature specialization in the training system of resident doctors and thus required all first-year resident doctors to undergo Postgraduate Year program training.

A general survey of the practices employed by many developed countries to tackle the challenge of changes in disease patterns in the new century indicates that medical students are required to receive one to two years of “general medical training” after graduation to enable them to acquire the ability to practice independently[3]. There have recently been calls for establishing a core competence-oriented training system for resident doctors, which includes patient-centered medical treatment, interdisciplinary collaborative work, professional practice based on evidence based medicine, improvements in medical treatment quality, and the utilization of information technology.

The content of the curriculum for the Postgraduate Year General Medicine Training Program was planned considering as reference the core competencies the country and society expected of doctors, with the six core competencies that doctors should possess proposed by the Accreditation Council for Graduate Medical Education (ACGME) in the USA, and the PGY general medical training that Taiwanese graduates should receive[4]. After several rounds of study and discussions among scholars and experts, a core curriculum for the one-year PGY training was formulated. It is now planned according to symptoms or signs, pathology, or disease while the teaching method centers on developing practical care for patients. In addition, clinical teaching and instructional methods are used as supplements to assist students in acquiring related knowledge. However, written tests or assignments are still relied upon to assess the knowledge and effectiveness of the training of doctors-in-training, thus resulting in passive and negative attitudes toward learning.

Thus, we employed new technology in the service of teaching and constructed relevant teaching methods. With newer developments in digital technology in the 21st century, the classroom teaching skills have become increasingly significant in the education. The continuous innovations in various kinds of digital technology have rendered the current teaching methods much more diverse than those of the past. Teaching methods have undergone immense changes. The “flipped classroom” method that has been widely discussed in recent years is a new teaching method that has attracted the attention of educators worldwide. As its name suggests, the core concept of the flipped classroom is to “flip” the teaching model; it aims to change the traditional teaching model, in which “teachers teach the content of the curriculum while students discuss and practice after lessons and then complete assignments,” to a newer way of attending class, in which “students watch videos of the curriculum content prepared by their teachers for them, engage in discussions and practices in class, and then complete their assignments” [5]. In both European and American teachers’ networks, the flipped classroom has been one of the most fervently searched keywords in the past few years and has been implemented in many classrooms today because the maturity of modern science and technology has made the production of teaching videos remarkably easy[6,7]. In addition, there are already significant video resources on YouTube or other websites that can be utilized. Secondary school teachers have proposed an idea that calls for allowing students to watch the teaching videos at home and using class time for exercises, experiments, group discussions, asking questions, summarizing main points, or other in-depth learning activities [8].

The aim of the flipped classroom is to change students’ learning mode from “passive learning” to “active learning” and to set the teaching objectives to higher levels of thinking skills such as analysis, integration, and argumentation[9].

Therefore, we employed the flipped classroom in the teaching of the core curriculum. We used actual clinical cases in teaching students how to learn. To confirm students’ learning outcomes, we specially designed a core curriculum that suited the flipped classroom model; students were required to analyze the cases, integrate information, and apply their knowledge in class while teachers observed their reactions and provided reinforcement and individual guidance. Through this model, we hope to truly attain our goals of cultivating new doctors with independent thinking and independent practice skills, and thus improve the quality of medical services and ensure patients’ safety.

To assess the training based on the core curriculum through the flipped classroom, we introduced the theory of reasoned action[10,11]. By analyzing the questionnaire, we delved into the important factors that affected students’ participation in their learning in the flipped classroom and the potential explanatory power of analysis through the theory of reasoned action.

## Materials and Methods

Using purposive sampling, 39 PGY students receiving training from August 2013 to July 2014 in the General Surgery of the Tri-Service General Hospital were selected as the participants of this study. The clinical teacher uploaded the core curriculum slides, unit teaching videos, and related learning information to the hospital intranet to assist students in preparing before class; this was followed by class discussions to foster a thinking mode of active learning. Two-way assessments were conducted during the course and multidimensional assessments and satisfaction surveys were administered after the course; the results were compared with the PYG students of the previous year to evaluate the learning outcomes of the students learning under the flipped classroom model. The control group included 43 students of the previous year, that is, the year before the intervention of the flipped classroom.

**Fig 1:**
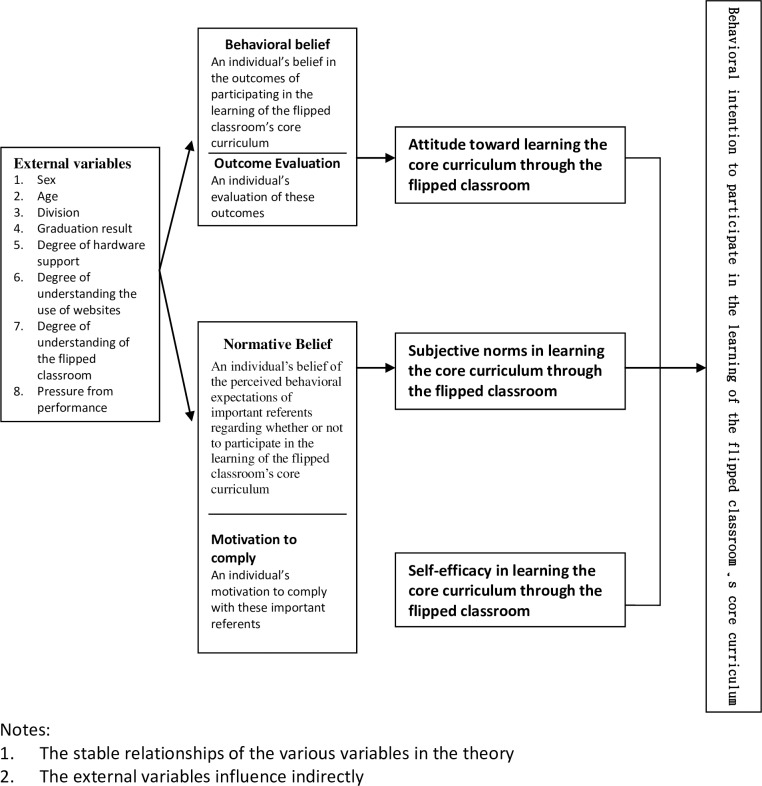
Research framework.

This study analyzed the outcomes of PGY students’ cognition of and persistent behavior in learning through the intervention of the flipped classroom. The theory of reasoned action was considered to design the study’s theoretical foundation (Fig 1).

The theory of reasoned action, developed by Fishbein and Ajzen in 1967, enables the prediction of individual behaviors, attitudes, and intentions. It has its theoretical base in social psychology, which considers an overall view of the dependent relationship between attitude, intention, and behavior. Through continuous development and verification, Fishbein and Ajzen proposed the notion of subjective norms and constructed a complete framework of the four in 1980[10,11]. Thus, the theory of reasoned action maintains that behavioral intention, the decisive factor directly affecting an individual’s actions, will be influenced by “attitude” and “subjective norms.” As for the behavioral influence produced by other factors, they can all indirectly influence behavior through behavioral intention. Therefore, when an individual’s attitude toward behavior is more positive, his behavioral intention will be higher and when his attitude toward behavior is more negative, his behavioral intention will be lower.

According to the theory of reasoned action (Fishbein & Ajzen, 1975), behavioral change begins with a change in an individual’s belief; in other words, if an individual believes that he/she should perform a certain behavior, this behavior will occur. Belief represents an individual’s volitional control over his/her behavior, thus indicating that the production of behavior is itself a behavior performed by an individual after making a decision on whether or not to perform [a behavior] or depending on other decisions based on his/her belief formed through logical thinking. This theory assumes that an individual will not be influenced by external environments; thus, a supportive thought can represent an individual’s behavior.

Ajzen and Fishbein (1980) defined an individual’s attitude toward performing a certain behavior as the possibility and good evaluation of the probable outcomes of the particular behavior. Thus, attitude can be explained in terms of two dimensions: behavior belief and outcome evaluation. An individual’s awareness of the social pressure involved in whether or not to perform a particular behavior is a function of normative belief and motivation to comply: Normative belief refers to an individual’s belief of the perceived behavioral expectations of important referent objects. Motivation to comply refers to an individual’s motivation to comply with these important referent objects.

The questionnaire for this study was designed following Fishbein and Ajzen’s (1980) method, as they were the first to use an open-ended questionnaire to sift out significant beliefs.

We compiled the questionnaire based on the significant beliefs acquired through the open-ended questionnaire, and after consulting related literature within and without Taiwan and discussing the findings with professor Chang. and Chen. Who takes in charge of PGY training program for more than 10 years.

The questionnaire included basic information on behavioral intention, attitude, subjective norms, behavior belief, outcome evaluation, motivation to comply, normative belief, self-efficacy, and external variables.

We revised the first draft of the questionnaire based on the experts’ comments. To know the participants’ understanding of the questions in the questionnaire, their reactions to answering it, the circumstances of the actual visits, and matters demanding attention and to assess the time required for answering the questionnaire, we selected eight to ten students from each year through stratified random sampling. Forty participants took the test, with 35 valid questionnaires being collected. The pretest process simulated the formal test in which the students were encouraged to raise questions and point out questions whose meanings were obscure or difficult to answer; these were recorded to enable us to understand the difficulties students faced when answering the questionnaire. The pretest results indicated that 25 mins were required to complete the questionnaire.

After collecting the questionnaires, Cronbach’s α was immediately applied to test the reliability of each scale; these results are shown in Table 1.

**Table 1:** Analysis of the internal consistency of the various scales in the pilot test (No. of people: 40).

Name of Scale	No. of Questions	Cronbach α
Knowledge	9	0.6763*
Bi*Ei	9	0.7888
Attitude	4	0.8544
NBj*MCj	8	0.8807
Subjective norms	4	0.7637
Self-efficacy	11	0.7353

Note

*Kuder-Richardson reliability.

As shown in Table 2, the questionnaire was subsequently finalized after making appropriate revisions based on pilot test result analysis, the test takers’ opinions on answering the questionnaire, and the item analysis results.

**Table 2:** Analysis of the internal consistency of the various scales in the formal test.

Name of Scale	No. of Questions	Cronbach α
Knowledge	6	0.503*
Bi*Ei	9	0.8689
Attitude	4	0.8458
NBj*MCj	8	0.6579
Subjective norms	4	0.8499
Self-efficacy	11	0.8688

Note

*Kuder-Richardson reliability.

After collecting the test data from the formal questionnaire, we coded, collated, and stored the data in a computer in the form of data files. Statistical analysis was conducted using SPSS for Windows. Cronbach’s α and Kuder-Richardson reliability were used to test all sub-scales and their reliability, which ranged between 0.6579 and 0.8689, thus indicating good internal consistency among all the scales and sub-scales in this questionnaire.

The study methods were reviewed and approved by the Institutional Review Board II of the Tri-Service General Hospital, National Defense Medical Center. U.S. regulations identify several research categories that are considered exempt from IRB oversight. The study matched the guide of research in conventional educational settings, such as those involving the study of instructional strategies or effectiveness of various techniques, curricula, or classroom management methods. In the case of studies involving the use of educational tests, there are specific provisions in the exemption to ensure that subjects cannot be identified or exposed to risks or liabilities.

The protocol was approved by the Hospital Institutional Review Board, and informed consent was deemed unnecessary (TSGHIRB No: 2-105-05-113).

## Results

With respect to the participants’ basic information and the sex distribution within the two groups, male students accounted for 64.6% (53 people) while female students accounted for 35.4% (29 people). For the experimental group learning the core curriculum through the flipped classroom, the average score on graduation was 88.2 ± 4.9 while for the control group learning through the traditional teaching method, it was 87.3 ± 5.4; thus, no statistical difference was observed in the two groups. The average age for the experimental and control groups was 26.9 ± 0.9 and 26.5 ± 1.1, respectively, which was also not a statistically significant difference. In terms of the distribution of divisions in the experimental and control groups, the number of students from general medicine was 8 and 9, respectively; from general surgery, 8 and 8, respectively; and from other divisions, 23 and 26, respectively; again, no statistically significant difference was noted Table 3 outlines these results.

**Table 3:** The characteristics of students in these two groups.

Variable	Category	No. of People in the Control Group 43 (Percentage)	No. of People in the Experimental Group 39 (Percentage)	P-value
**Sex**				0.648
	Male	28 (65.1%)	25 (64.1%)	
	Female	15 (34.9%)	14 (35.9%)	
**Age**				0.786
		26.5 ± 1.1	26.9 ± 0.9	
**Average result on Graduation**				0.824
		87.3 ± 5.4	88.2 ± 4.9	
**Division**				0.791
	General Medicine	9	8	
	General Surgery	8	8	
	Other Divisions	26	23	

The correlation between the variables in the theoretical model of reasoned action and the intention of participating in learning the core curriculum through the flipped classroom reached the significance level. The correlation between self-efficacy and behavioral intention was the highest (r = .491, P < .01), followed by attitude (r = 0.365, P < 0.01) and subjective norms (r = 0.360, P < 0.01). All three showed positive correlations with behavioral intention; among attitude, subjective norms, and self-efficacy, the pairwise correlations also reached the significance level (Table 4).

**Table 4:** The correlation matrices of intention, attitude, subjective norms, and self-efficacy in the learning of the core curriculum through the flipped classroom.

Variable	Intention	Attitude	Subjective norms	Self-efficacy
Intention	1.00			
Attitude	0.365**	1.00		
Subjective norms	0.360**	0.454**	1.00	
Self-efficacy	0.491**	0.370**	0.355**	1.00

Note

**P < 0.01.

Furthermore, by including self-efficacy in the regression model with attitude and subjective norms, it was revealed that self-efficacy had the greatest influence on behavioral intention (β = 0.385, P < 0.001) while the effects of attitude and subjective norms on behavioral intention were identical (β = 0.155, P < 0.01) (Table 5).

**Table 5:** Multiple regression analysis of the intention in the learning of the core curriculum through the flipped classroom.

Step	Independent variable	ß	R	R^2^	F	R^2^ change	F-change
STEP1	Attitude	0.257					
	Subjective norms	0.246	0.427	0.183	43.708**		
STEP2	Attitude	0.155					
	Subjective norms	0.155					
	Self-efficacy	0.385	0.551	0.304	56.609**	0.121	56.609**

Note

**P < 0.01.

**Fig 2:**
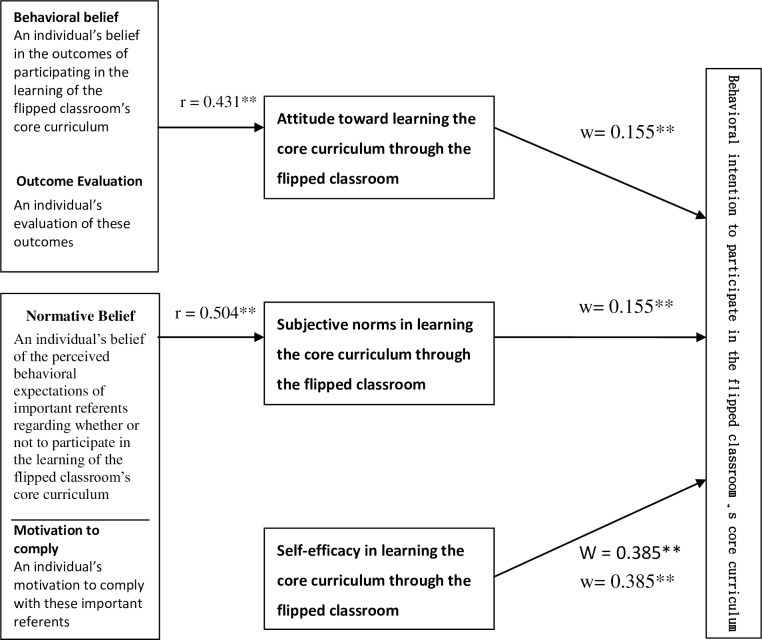
Diagram of the relationships of various variables.

This study mainly focused on employing the theory of reasoned action to predict and explain the participants’ behavioral intention to participate in learning the core curriculum through the flipped classroom. The following points attempt to synthesize the relationship of the various key variables (Fig 2) to verify whether they correspond with this study’s theoretical viewpoints and investigate the theory’s suitability for studying the behavioral intention to participate in learning the core curriculum through the flipped classroom. The findings are as follows:

1. The correlation of attitude, subjective norms, and self-efficacy with the behavioral intention to participate in the learning of the core curriculum through the flipped classroom reached significance level; learning intention was most correlated with the participants’ self-efficacy while its correlations with subjective norms and attitude were identical.
2. With regard to behavior belief, outcome evaluation, normative belief, and motivation to comply, the participants reached overall significant differences.
3. The sum of the cross products of attitude and behavior belief was significantly and positively correlated with outcome evaluation and that of subjective norms and normative belief with motivation to comply also showed the same correlation.
4. The study supports the theory of reasoned action: students’ attitudes and subjective norms in performing a certain behavior were the two main factors affecting their behavioral intention; the effect can be further enhanced if self-efficacy is added to attitude and subjective norms.

Moreover, after the intervention of the learning of the core curriculum through the flipped classroom, these students were assayed of daily clinical works and basic tests by clinic physicians, and significant differences were observed in the knowledge and attitudes of the students of the experimental group. They became more independent in their learning, their attitude assessment (360° evaluation, six core competencies) indicated greater mutual cooperation, and they showed more confident in their work (Table 6).

**Table 6:** Differences in the students with respect to knowledge, skills, and attitude before and after the intervention of the learning of the core curriculum through the flipped classroom.

Variable	Before intervention (Mean ± Standard deviation)	After intervention (Mean ± Standard deviation)	p-value
Knowledge			P < 0.01*
	89.42 ± 3.85	92.68 ± 3.46	
Skills (OSCE)			0.460
	85.07 ± 4.02	85.95 ± 6.67	
Attitude 360			P < 0.01*
	81.14 ± 1.81	82.94 ± 1.46	

Note

*P < 0.01.

OSCE (Objective Structured Clinical Examination).

## Discussion

Today, the flipped classroom has been used in different areas of teaching. Within the flipped classroom model, students watch videos at home as homework while teachers use the class time for conducting discussions among students under their guidance. Several years ago, Sal Khan and YouTube shared a very futuristic flipped classroom model, and today it has been discovered that the flipped classroom can even lead to a 5% increase in students’ overall results[7]. This finding highlighted that students learning the curriculum delivered through a flipped classroom generally outperform those learning one delivered through traditional instructional methods [12]. Along with the increasing popularization of tablets, many teachers tend to let their students prepare at home, thus allowing students to achieve better learning outcomes at their own learning pace and reserving more class time for further in-depth dialogue and interaction.

The flipped classroom has currently attracted people’s attention and is being implemented as a result of its timely ride on the popularity of online e-teaching, especially the effect of massive open online courses (MOOCs), which have impressively developed into the new digital learning trend[13].

Although the flipped classroom is highly valued and has been practiced by teachers in schools of various levels, very few attempts have been made until now to report the learning outcomes achieved through the flipped classroom by means of rigorous research methods[14].

Goodwin and Miller stated that the flipped classroom has grown rapidly, but very little research has been conducted on it [15]. Bishop and Verleger also suggest and hope that more studies using controlled experiments or quasi-experimental designs in the investigation of learning outcomes will be conducted; they also hope that teachers will integrate theoretical foundations when designing classroom activities [14]. Therefore, we employed the flipped classroom to conduct core curriculum training. We included the theory of reasoned action and, through the analysis of the questionnaire, delved into the important factors that affected students’ participation in the learning in the flipped classroom and the explanatory ability of the possible analysis made in consideration of the theory of reasoned action.

The theory of reasoned action, proposed as early as 1967 after years of improvement and verification, was finally co-developed by Ajzen and Fishbein. It aimed to understand and predict individual behaviors. The theory’s fundamental premise is that humans are “rational” individuals and will therefore first consider a particular behavior and its consequences before performing it and only then will they decide whether or not to act. Thus, the theory of reasoned action has two hypotheses: 1. most human behaviors are under their own volitional control and also in accordance with reason and 2. the behavioral intention that determines whether or not humans perform a certain behavior is the immediate determinant of the occurrence or non-occurrence of that behavior.

Self-efficacy is also an important variable in changing and maintaining behavior. It is one of the core concepts of Bandura’s social learning theory [10,11]. Self-efficacy refers to an individual’s belief in his/her ability to perform a specific behavior in a specific situation; it can serve as a predictive variable when explaining the individual’s extent of behavior. The source of this predictive variable varies in its performance level and strength according to the variables of cognition, sociality, and environments, thus resulting in different behavioral outcomes.

The theory of self-efficacy has been widely used in the study of health behaviors, and therefore, many scholars have applied this theory to elicit and predict health behaviors [16–19].

Using the theories of reasoned action and self-efficacy, our study mainly aimed to predict and explain participants’ behavioral intention in participating in core curriculum training through the flipped classroom. It synthesized the relationships of the various key variables (Table 3) to verify whether they correspond to this study’s theoretical perspectives and investigated the suitability of applying the theory to study the behavioral intention to participate in the core curriculum training through the flipped classroom.

Besides focusing on the students’ attitude toward and subjective norms in participating in the core curriculum training through the flipped classroom, this study also examined self-efficacy; based on these four aspects, we explained the variance of individuals’ behavioral intention to participate in the training. The result indicated that the ability to explain the variance of behavioral intention to participate in the core curriculum training through the flipped classroom was enhanced when self-efficacy was included. This result reached significance level.

Vries et al. asserted that self-efficacy can be added to attitude and subjective norms to integrate the theory of reasoned action with the self-efficacy of the social learning theory, which can then enhance the ability to explain behavior or behavioral intention [20]. The results of this study also support this assumption.

Furthermore, the correlation value of the sum of the cross products of attitude, behavioral belief, and outcome evaluation was 0.360 while that of subjective norms and motivation to comply was 0.510, both reaching significance level (p < 0.01). These findings support the views in the theory of reasoned action that attitude is shaped by the sum of the cross products of behavioral belief and outcome evaluation while subjective norms are shaped by the sum of the cross products of normative belief and motivation to comply.

Therefore, the results of this study confirm that it is possible to include self-efficacy in the theory of reasoned action to concretely explain the changes in behavioral intention with respect ot participation in the core curriculum training through the flipped classroom.

At present, the teaching model of the flipped classroom has been a focal point in teaching reform. Under this model, teachers can see the growth of their students as they become more independent in their learning, show stronger motivation and more responsibility for their own learning, and transform into more active learners [21,22].

**Fig 3:**
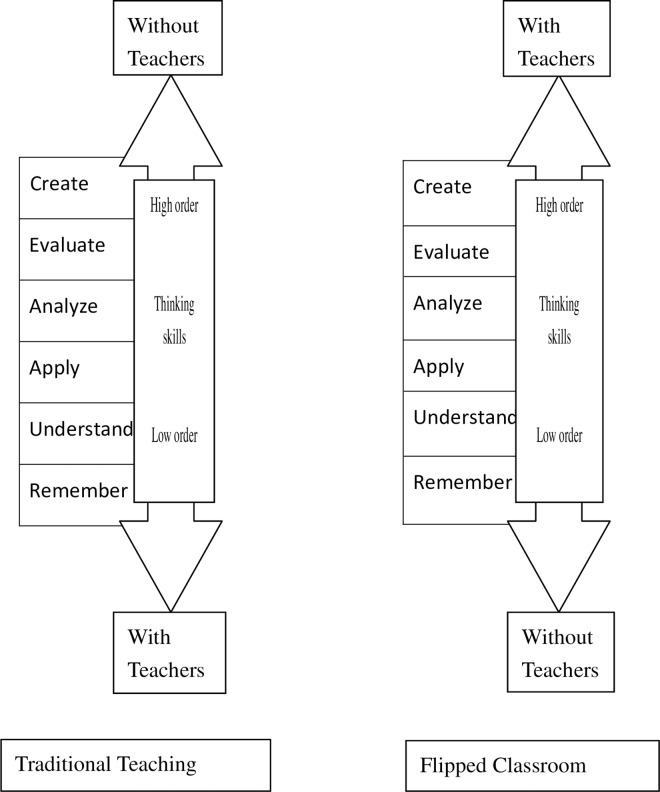
Educational Objectives of Bloom’s Cognitive Domain.

Active learning opportunities in class increase in the flipped classroom, which serves to facilitate students’ learning and achievements [23]; students can learn to be attentive and think critically, thus improving their learning attitude [24]; before-class preparation helps in students’ interaction and performance in class, thus enabling them to manage their internal cognitive loads and in turn facilitating their learning [25,26]; using the time before class to understand and remember the teaching contents creates room for more peer interaction and teaching in class, thus increasing the opportunities for students to learn higher-level cognitive abilities—apply, analyze, evaluate, and create—in the teaching objectives of Bloom’s cognitive domain (Fig 3) and making students more confident [27,28]. Active learning provides students with more opportunities and content interaction [29].

However, the flipped classroom’s success depends on students’ preparation before class. Walvoord and Anderson adopted the assignment-based model, which requires students to write a few questions before class to get feedback for the same in class [30]. Besides this, online interaction, key notes, and problems-recording are all possible methods. If students have not utilized their time before class to better understand and remember teaching contents, it indicates that they have not fulfilled their responsibility; attending class without learning the required knowledge will render in-class group assignments and activities ineffective. In other words, such a student can hardly become an independent and autonomous learner. The real strength of education lies in teaching students to engage in active inquiry and learning rather than the videos; students, in learning independently, must simultaneously be able to search the materials they need and critically review them. The core duty of the flipped classroom is developing a student-centered teaching method, thus the students will control and possess their own learning.

This study shows that by employing the teaching method of the flipped classroom in the PGY general medicine training, the students’ learning behavior could indeed be changed from the learning mode of “passive learning” to that of “active learning,” with better learning outcomes. Moreover, the theory of reasoned action can also be used completely to predict and explain students’ learning intention for the teaching method of the flipped classroom and their learning outcomes.

A limitation of this study was the scope of the intervention. Expanding the intervention to other modules or other courses, where appropriate, may provide additional evidence. Another limitation of the study was the modest although significant improvements in student performance.

## Conclusions

The flipped classroom model is gaining recognition in a wide variety of academic settings as an approach to promote students’ learning. This study is the first study showed the efficacy of flipped classroom in PGY students’ training program and proved by the theory of reasoned action. The implementation of the flipped classroom in this study accompanied improved student performance and generated positive student attitudes towards the experience. Of course, further research is needed to continue the investigation into the efficacy of the flipped classroom.
